# The Control of the Invalid Children's Aid Association

**Published:** 1914-01-03

**Authors:** 


### THE CONTROL OF THE INVALID CHILDREN'S AID ASSOCIATION.

All hospital almoners in London, and most of the out-patient physicians and surgeons, have good reason to thank the Invalid Children's Aid Association for the after-care of youthful patients who might otherwise fail to extract full benefit from their attendance at hospitals. Convalescent homes; sanatoria; financial aid in the purchase of surgical boots, instruments, and appliances; the loan of invalid carriages; clothes; extra nourishment; and many other forms of philanthropic assistance are provided by the I.C.A.A. for thousands of London children whose chances of obtaining them through any other agency would be infinitesimal.

There is no need to dilate upon the value of the work thus done, for every institutional worker who takes any interest in the welfare of child patients outside the four walls of the voluntary institutions themselves knows that it is very great. The progress and prosperity of this Association are therefore matters of moment, and recent events which are tending to alter its constitution in some respects demand some notice, for they may affect the scope and character of the Association's activities.

The I.C.A.A., it should be understood, consists of a Central Executive Council, which directly controls, finances, and administers the work in certain parts of London known as districts. Besides this there are a number (about thirty) of branches, each of which undertakes the responsibility of managing its own area, including the raising of funds. At the present time the funds raised, children provided for, and areas of London covered by the branches on the one hand, and the Central Council with its districts on the other, are fairly equal, roughly speaking. A few years ago the number of branches was much smaller, and the burden on the Central Council proportionately much greater. As more and more branches are formed, each relieving the Central Council of a portion of its work, a time will soon come when the branches between them will be doing the major part of the work. This seems to be quite certain, and is a process of evolution and devolution which is healthy, natural, and politic.

So far the branches have had no share whatever in the management of the Association, for this is vested solely in the Central Council. Occasionally it happens that the general policy of the latter does not commend itself to the branches, or to the greater number of them. But in such an event the branches are powerless to influence the Central Council, where their views are not considered in the least. The branches, accordingly, manifest from time to time irritation with the constitution which denies them any voice in controlling a society so large a proportion of whose work is done by them; and various attempts have been made towards gaining better representation and more influence. Amongst other things a conference of branches was established, consisting of delegates from each branch; this, it was hoped, would be able to impress the Central Council sufficiently to secure at least attentive consideration for its views. It has happened, however, that the Central Council takes no more notice of the conference's views than of the views of individual branches; and at its last meeting various measures were propounded for obtaining a remedy for what the branches regard as a grievance.

The Paddington Branch, for instance, brought forward a resolution which practically held a pistol at the head of the Central Council, for it was worded in an almost minatory tone. This motion was felt to be undiplomatic; it was much modified in details, and the peremptory note was softened down. As ultimately adopted, and communicated officially to the Council, it invites the latter to send six members to each conference, the latter to be held quarterly, or more often if necessary. The views of the branches will thus filter through to the Central Council by the medium of these six delegates; and not only the views, but the facts and the reasoning on which they are founded.

The scheme is a compromise it will be seen; and it remains to be seen how it will work. If the Central Council, with its autocratic powers and undemocratic constitution, abandons the attitude of aloofness with which it has sometimes exasperated the branches, the compromise may work well enough for a few years at least. But if it accepts the proposals of the conference merely as a convenient way of shelving reform and keeping the branches quiet for a while, the future of the Association may be gravely prejudiced. *Per contra*, the Council is, beyond doubt, well-intentioned; the experience of some of its members is much greater than that of most of those who take part in the management of branches, and we should be sorry to say that when disagreements occur the Council is always in the wrong. The truth would seem to be that some of the members of the Council are "deadheads," and that an infusion of new blood would do good. At all events, any schism or internal friction would produce widespread dismay,

